# miR155, TREM2, INPP5D: Disease stage and cell type are essential considerations when targeting clinical interventions based on mouse models of Alzheimer’s amyloidopathy

**DOI:** 10.1186/s12974-023-02895-7

**Published:** 2023-09-25

**Authors:** Sam Gandy, Michelle E. Ehrlich

**Affiliations:** 1 Department of Neurology, Icahn School of Medicine at Mount Sinai, New York, NY 10029 USA; 2 Department of Psychiatry and Alzheimer’s Disease Research Center, Icahn School of Medicine at Mount Sinai, New York, NY 10029 USA; 3 James J Peters VA Medical Center, Bronx, NY 10468 USA; 4 Department of Pediatrics, Icahn School of Medicine at Mount Sinai, New York, NY 10029 USA; 5 Department of Genetics and Genomic Sciences, Icahn School of Medicine at Mount Sinai, New York, NY 10029 USA

## Abstract

Studies of microglial gene manipulation in mouse models of Alzheimer’s disease (AD) amyloidopathy can cause unpredictable effects on various key endpoints, including amyloidosis, inflammation, neuritic dystrophy, neurodegeneration, and learning behavior. In this *Correspondence*, we discuss three examples, microRNA 155 (miR155), TREM2, and INPP5D, in which *observed* results have been difficult to reconcile with *predicted* results based on precedent, because these six key endpoints do not reliably track together. The pathogenesis of AD involves multiple cell types and complex events that may change with disease stage. We propose that cell-type targeting and timing of intervention are responsible for the sometimes impossibility of predicting whether any prospective therapeutic intervention should aim at increasing or decreasing the level or activity of a particular molecular target.

Studies of microglial gene manipulation in mouse models of Alzheimer’s disease (AD) amyloidopathy can cause unpredictable effects on various key endpoints, including amyloidosis, inflammation, neuritic dystrophy, neurodegeneration, and learning behavior. In this *Correspondence*, we discuss three examples, microRNA 155 (miR155), TREM2, and INPP5D, in which *observed* results have been difficult to reconcile with *predicted* results based on precedent, because these six key endpoints do not reliably track together (Table 1). miR155 is a microRNA in neurons and glia that modulates immune function and the response to viral infection [1–6]. TREM2 is a cell-surface protein in microglia that senses the presence of protein or lipid debris in the interstitial spaces of the brain [8–17, 19, 20]. INPP5D is a phospholipid and phosphoprotein phosphatase in microglia that regulates phagocytosis and clustering of microglia around amyloid plaques [21–25].

**Table 1:** Summary of confounding results from manipulations of miR155, TREM2, or INPP5D in various mouse models of Alzheimer’s pathology

Mouse	Amyloidosis	Inflammation	Dystrophy	Behavior
PSAPP X miR155 pancellular constitutive knockout [3]	Increased	Unchanged	N/A	Improved
PSAPP X miR155 microglia-specific inducible knockout (PS1^Δexon9^) [5]	Decreased	Increased	N/A	N/A
PSAPP X miR155 microglia-specific inducible knockout (PS1^L166P^) [6]	Increased compaction	Decreased	Decreased	Improved
5xFAD X microglial TREM2 inducible overexpression (early induction) [10]	Decreased early and mid-stage; no change late stage	Decreased; increased microglia around plaque	Decreased	N/A
5xFAD X microglial TREM2 inducible overexpression (mid-stage induction) [10]	Decreased early and mid-stage; no change, late stage	Decreased; increased microglial & dynamics around plaques	Unchanged	N/A
5xFAD X microglial TREM2 ^R47H^ inducible overexpression (early induction) [10]	Increased mid-stage; no change late stage	Decreased; decreased microglia dynamics around plaques; no change microglia number per plaque	Increased	N/A
5xFAD X TREM2 ^H157Y^ CRISPR knock-in [11]	Decreased	Decreased	Decreased	Enhanced LTP
5xFAD X AAV sTREM2 or sTREM2 protein [13]	Decreased	Increased	N/A	Improved
WT X AAV TREM2 [14]	N/A	Increased phagocytosis	N/A	Impaired
PSAPP X AAV TREM2KO (early-middle) [14]	Unchanged	Inhibited phagocytosis	N/A	Improved
PSAPP X AAV TREM2 KO (middle-late) [14]	Increased	Inhibited phagocytosis	N/A	Impaired
PSAPP X AAV TREM2 (early-middle) [14]	Decreased	Increased phagocytosis	N/A	Impaired
PSAPP X AV TREM2 (middle-late) [14]	Decreased	Increased phagocytosis	N/A	Improved
5xFAD or APPPS121 TREM2 constitutive knockout [15]	Decreased	Decreased	Decreased	N/A
PS2APP X TREM2 −/− constitutive knockout (6–7 months, females) [16]	Increased	Decreased	Increased	N/A
PS2APP X TREM2 −/− constitutive knockout (12, 19–22 months, both sexes) [16]	Decreased	Decreased	Increased	N/A
PS2APP X TREM2 (−/−) vs (+/+) [16]	Intermediate Decrease	Intermediate effect on microglial clustering	Increased	N/A
APP knock-in + TREM2 activating antibody [17]	Decreased	Increased phagocytosis	N/A	N/A
APP knock-in + TREM2 blocking antibody 17	Decreased	Decreased	N/A	N/A
5xFAD + TREM2 activating antibody (chronic) [19]	Unchanged	Migration toward plaques	Increased	N/A
TREM2 KO pancellular knockdown (−/+) vs (−/−) [20]	N/A	(−/+) Increased; (−/−) protected	(−/+) Increased; (−/−) protected	N/A
PSAPP X INPP5D microglial knockout [21]	Increased	Increased	N/A	N/A
5xFAD X INPP5D microglial knockout [22]	Unchanged	Increased	Increased	N/A
APP ^NL-G-F/NL-G-F^ X INPP5D microglial knockout [23]	Unchanged	Unchanged	Unchanged	N/A
5xFAD X INPP5D pancellular knockout [24]	Decreased	Decreased	N/A	Improved

MicroRNAs are important regulators of many facets of physiological function while also being implicated in the pathogenesis of several systemic and neurological disorders, including immunity, inflammation, viral infection, cancer, cardiovascular disease, AD, and Down syndrome (for review, see ref. [1]). Elevation of miR155 levels in human AD brain was recently confirmed and localized to microglia and hippocampal neurons [2, 3], observations that were extended to an AD amyloidopathy mouse model [4]. These data made miR155 an attractive target for controlling neuroinflammation. However, three recent papers raise the possibility that therapeutic modulation of miR155 might not be straightforward [3, 5, 6]. In a 2020 study [3], APP/PS1 mice on a constitutive pancellular *miR155* knockout background showed improvement in synaptic function and learning behavior despite an *increase* in amyloid burden. In one of the 2023 studies, selective and conditional deletion of *miR155* from microglia in pre-symptomatic APP/PS1 mice caused new seizures to appear despite a *decrease* in amyloid burden [5]. In the other 2023 study [6], also using an inducible microglia KO strategy and APP/PS1 mice, the result was increased plaque compaction, reduced neuritic dystrophy, and improved behavior. Both 2023 papers used inducible, microglia-specific knockouts, and both gave tamoxifen at 6–8 weeks of age yet the phenotypes were different, perhaps because different PS1 mutations were used: in the former, the exon 9 deletion PS1 mutation was used [5], while the other used the L166P point mutation [6]. While we are unable to explain the conflicting data and complex effects, we note that such dramatic and irreconcilable differences are not unusual for studies in mouse models of AD amyloidopathy [7]. In these studies, key endpoints of amyloid burden, inflammation, neuritic dystrophy, neurodegeneration, and learning behavior are not consistently linked. We propose that the targeted cell type and timing of intervention can cause dramatic shifts in amyloid burden, microglial function, inflammation, and learning behavior and their relationships to one another.

Another dramatic example of discrepant results occurs with experimental manipulation of TREM2 (*t*riggering *re*ceptor on *m*yeloid cells-*2*). R47H is a well-known *TREM2* mutation that increases the risk for AD [8, 9]. The molecular pathogenesis of this mutation is believed to relate to the inability of the mutant TREM2 to play its usual roles in modulating neuroinflammation and phagocytosis of Aβ. R47H shifts both endpoints in a damaging direction, leading to increased neuroinflammation and amyloid burden. Based on these data, it was initially hypothesized that TREM2 loss-of-function would increase pathology in mouse models, and that increased activity would mitigate pathology. The effects of the R47H mutation were confirmed following conditional overexpression of TREM2 R47H in mouse models, in which the mutant TREM2 transgene causes amyloid burden to increase [10]. These mutation-related effects contrast with those from *TREM2*^*H157Y*^, a genetic variant that also increases risk for AD; yet, H157Y *reduces* amyloidosis in a mouse model, suggesting that this mutation increases risk for AD by acting through an amyloidosis-independent pathway [11].

Since loss-of-function was predicted to exacerbate phenotype, increased TREM2 was predicted to ameliorate phenotype. In fact, constitutive or conditional overexpression of wild-type TREM2 is beneficial prior to amyloid deposition in models of early AD; yet, when TREM2 is overexpressed after amyloidosis is established, both amyloid burden and neuritic dystrophy are exacerbated [10]. Alternatively, when the 5xFAD mutations are on a constitutive *Trem2* knockout background, accumulation of Aβ peptides is enhanced both within neurons and in the interstitial extracellular compartment [12]. After discovery of pathogenic mutations in the *TREM2* gene, metabolism of the transmembrane holoprotein by α-secretase was discovered to release the TREM2 ectodomain as a protective factor known as soluble TREM2 (sTREM2), and its effects in mouse models have also been assayed. Experimental viral transduction with TREM2 and sTREM2 complicated matters further. In one study, adeno-associated virus (AAV) expressing sTREM2 was injected into symptomatic, 7-month-old 5xFAD mice, resulting in proliferation of microglia, reduction in amyloid burden, and preservation of normal learning behavior [13]. Viral overexpression of TREM2 in the hippocampi of wild-type mice resulted in significant synaptic impairment [14]. The same report showed that TREM2 overexpression enhanced microglial phagocytosis of synapses during the early-to-middle stage of amyloidosis. However, TREM2 overexpression and upregulated microglial phagocytosis came to play positive roles by the middle-to-late stage of pathology when TREM2 overexpression reduced amyloid deposition [14].

Genetic deletion of *Trem2* exerted different outcomes depending on the transgenic line, timing of the intervention and the age of the mouse [15]. Constitutive deletion in APP/PS1 mice led to decreased amyloid accumulation [15]. Knockdown of *Trem2* at the early-to-middle stage of amyloid accumulation (2–6-month-old APP/PS1 mice) prevented synaptic phagocytosis by microglia, whereas knockdown of *Trem2* at the middle-to-late stage (6–10-month-old APP/PS1 mice) caused impairment of microglial phagocytosis, exacerbated amyloid burden, and destroyed synapses via amyloid toxicity [15]. On the other hand, deletion in PS2APP mice led to increased amyloid accumulation in 7-month-old female mice, but reduced accumulation in both sexes by 21–22 months, whereas neuritic dystrophy was increased [16].

More examples of divergent results have arisen from modeling of therapeutic intervention using anti-TREM2 antibodies. Antibody 4D9 blocks shedding of sTREM2, activates protective TREM2 signaling, and improves pathology in a mouse model of AD amyloidopathy [17]. Agonistic antibodies such as 4D9 and others are currently being tested in clinical trials [17, 18]. It is worth noting that while TREM2-activating antibodies show promise in reducing amyloid burden at early stages, if treatment is continued into later stages of pathology, TREM2 agonism may exacerbate seeding and spreading of tauopathy [19]. This means that for the TREM2-activating antibody to be useful as a long-term therapy, it is likely that complete purging of amyloid (e.g., with one of the anti-amyloid antibodies) will be required prior to initiation of TREM2-activating antibody treatment. TREM2 and tauopathy represent another interaction with conflicting results. Both TREM2 activation and TREM2 inhibition have been reported to attenuate tauopathy [19, 20]. Finally, the level of TREM2 downregulation also led to unexpected results, in that genetic haploinsufficiency vs total deletion led to opposite effects on tauopathy rather than causing similar effects in a dose-dependent fashion [20].

Studies on the role of INPP5D in amyloidosis studies have likewise yielded a mixture of results. *INPP5D* is another microglial AD risk gene identified by GWAS, and four recent *Inpp5d* knockdown studies in cerebral amyloidosis models were each slightly different in design, and each showed different effects [21–24]. Despite disparate impact on amyloid burden, three related results were similar across several *Inpp5d* knockdown studies: (1) increased plaque-associated microglia; (2) increased microglial barrier; and (3) attenuated neuritic dystrophy. Castranio *et* *al*. selectively and inducibly knocked down *Inpp5d* in the microglia of early symptomatic APP/PS1 mice crossed with floxed *Inpp5d* mice, using the CX3CR1-Cre mouse; this genotype and treatment exacerbated the amyloid burden and increased expression of disease-associated microglial genes[21]. A similar study by Samuels *et al.* used the same approach to target microglial *Inpp5d* in 1 month-old 5xFAD mice, but, in those mice, amyloid burden was unchanged by the *Inpp5d* deficiency[22]. Iguchi *et al. *(2023) studied the effects of *Tyrobp* deficiency and *Inpp5d* deficiency (singly and in combination) on the *App*^*NL−G−F/NL−G−F*^ knock-in mouse[23]. As with the Samuels *et al.* [22] study, *Inpp5d* deficiency alone had no effect on amyloid burden in the Iguchi *et al.* study [23]. In the fourth study, Lin *et al.* [24] used a constitutive pancellular *Inpp5d* knockdown approach and reported increased plaque clearance and reduced amyloid burden in the presence of *Inpp5d* haploinsufficiency. Taken together, these papers suggest that modulation of amyloid burden may not be the most important effect of alterations in INPP5D, which apparently differs by stage of disease. This implies that clinical trials of INPP5D inhibitors might show cognitive benefits even if amyloid burden is unchanged or increased. Further complicating the miR155 and INPP5D data interpretation is that INPP5D is a direct target of miR155, and is increased twofold in the brain in the absence of miR155 [25].

Our point is not that some results are right, and some are wrong, but rather that the pathogenesis and staging of AD are so complex that it can be challenging to determine whether increasing or decreasing the level or activity of a target is more likely to cause benefit and that the beneficial direction may even change with the targeted cell type or disease stage. The two *miR155* knockout studies and the multiple TREM2 and INPP5D studies provide examples for how disease stage and/or cell type can differentially alter the outcome as well as examples where cognitive benefit does not track with amyloid burden. Defining this complexity in mouse models enables us to take these issues into account when we design human clinical trials.

## Data Availability

All data sets are available upon request.

## Abbreviations

AAV: Adeno-associated virus
AD: Alzheimer’s disease
hiPSCs: Human-induced pluripotent stem cells
INPP5D: Inositol polyphosphate-5-phosphatase D
TREM2: Triggering receptor on myeloid cells-2
