# Correction: miR155, TREM2, INPP5D: Disease stage and cell-type are essential considerations when targeting clinical interventions based on mouse models of Alzheimer’s amyloidopathy

**DOI:** 10.1186/s12974-023-02954-z

**Published:** 2023-11-30

**Authors:** Sam Gandy, Michelle E. Ehrlich

**Affiliations:** 1 Department of Neurology, Icahn School of Medicine at Mount Sinai, New York, NY 10029 USA; 2 Department of Psychiatry and Alzheimer’s Disease Research Center, Icahn School of Medicine at Mount Sinai, New York, NY 10029 USA; 3 James J Peters VA Medical Center, Bronx, NY 10468 USA; 4 Department of Pediatrics, Icahn School of Medicine at Mount Sinai, New York, NY 10029 USA; 5 Department of Genetics and Genomic Sciences, Icahn School of Medicine at Mount Sinai, New York, NY 10029 USA

**Correction: Journal of Neuroinflammation (2023) 20:214** **https://doi.org/10.1186/s12974-023-02895-7**

In this article, the paragraph beginning with “More examples of….” were corrected. The corrected papragraph is given below.

More examples of divergent results have arisen from modeling of therapeutic intervention using anti-TREM2 antibodies. Antibody 4D9 blocks shedding of sTREM2, activates protective TREM2 signaling, and improves pathology in a mouse model of AD amyloidopathy [17]. Agonistic antibodies such as 4D9 and others are currently being tested in clinical trials [17, 18]. It is worth noting that while TREM2-activating antibodies show promise in reducing amyloid burden at early stages, if treatment is continued into later stages of pathology, TREM2 agonism may exacerbate seeding and spreading of tauopathy [19].

The original article has been corrected.
